# Cardioversion of atrial fibrillation does not affect obstructive sleep apnea

**DOI:** 10.1080/03009734.2017.1291545

**Published:** 2017-03-14

**Authors:** Niklas Höglund, Carin Sahlin, Milos Kesek, Steen M. Jensen, Karl A. Franklin

**Affiliations:** a Public Health and Clinical Medicine, Heart Centre, Umeå University, Umeå, Sweden;; b Public Health and Clinical Medicine, Medicine, Umeå University, Umeå, Sweden;; c Surgical and Perioperative Sciences, Surgery, Umeå University, Umeå, Sweden

**Keywords:** Atrial fibrillation, cardioversion, polysomnography, sleep apnea

## Abstract

**Background:**

Sleep apnea is common in patients with atrial fibrillation, but the effect of the cardioversion of atrial fibrillation to sinus rhythm on central and obstructive apneas is mainly unknown. The primary aim of the study was to analyze the association between cardioversion of atrial fibrillation and sleep apneas, to investigate whether obstructive or central sleep apneas are reduced following cardioversion. A secondary objective was to study the effect on sleep quality.

**Methods:**

Twenty-three patients with atrial fibrillation were investigated using overnight polysomnography, including esophagus pressure monitoring and ECG, before and after the cardioversion of persistent atrial fibrillation.

**Results:**

Obstructive sleep apnea occurred in 17/23 patients (74%), and central sleep apnea in 6/23 patients (26%). Five patients had both obstructive and central sleep apnea. Sinus rhythm at follow-up was achieved in 16 patients. The obstructive apnea-hypopnea index, central apnea-hypopnea index, and the number of patients with obstructive or central sleep apnea did not differ before and after restoration of sinus rhythm. Sleep time, sleep efficiency, time in different sleep stages, and subjective daytime sleepiness were normal and unaffected by cardioversion.

**Conclusions:**

Both obstructive and central sleep apneas are highly prevalent in patients with persistent atrial fibrillation. Obstructive sleep apneas are unaffected by the cardioversion of atrial fibrillation to sinus rhythm. The sleep pattern is normal and unaffected by cardioversion in patients with atrial fibrillation.

**Clinical Trial Registration:**

Trial number NCT00429884.

## Introduction

Atrial fibrillation is common and increases with age. It occurs in about 6% after the age of 55 and in 14% after the age of 80 (1). Subjects with atrial fibrillation run an increased risk of stroke, heart failure, and early death (2,3). Obstructive sleep apnea with repetitive apneas during sleep is another common condition that implies an increased risk of early death, hypertension, stroke, and atrial fibrillation (4–12). Male gender, obesity, and age are risk factors for both atrial fibrillation and obstructive sleep apnea. Obstructive sleep apnea is common in patients with atrial fibrillation, and atrial fibrillation is common in patients with central sleep apnea (13–17).

The effect of cardioversion of atrial fibrillation on obstructive and central sleep apnea is, however, mainly unknown. The primary aim of the study was to analyze the association between cardioversion of atrial fibrillation and sleep apneas, to investigate whether obstructive or central sleep apneas are reduced following cardioversion. A secondary objective was to study the effect on sleep quality.

## Materials and methods

### Patient population and trial design

The study comprised 28 patients scheduled for elective cardioversion of persistent atrial fibrillation at the Department of Cardiology, Umeå University Hospital, without previously diagnosed sleep apnea. The Regional Ethics Committee at the medical faculty at Umeå University approved the study protocol, and written informed consent was obtained from all the participating patients. Five patients were subsequently excluded, due to severe heart failure, unstable angina pectoris, or withdrawal of consent.

The patients were included two to four weeks prior to the cardioversion. A detailed medical history was obtained, a 12-lead electrocardiogram (ECG) was performed, and the patients underwent an echocardiography. Overnight polysomnography was performed within one week prior to the cardioversion. A follow-up polysomnography and ECG was done within one week after cardioversion.

Cardioversion was performed on an elective outpatient basis. Sedation was induced by propofol (Diprivan^®^) 1 mg/kg i.v. administered by an anesthesiologist. R-wave-synchronized mono- or biphasic anterio-posterior shocks were given in a step-up protocol at 200 J, 300 J, and 360 J to achieve sinus rhythm.

Overnight polysomnography (Embla, Flaga hf, Iceland) was performed in hospital at 20 °C/68 °F and included continuous recordings of electroencephalograms (C3-A2, C4-A1), electro-oculograms, submental electromyograms, airflow with a three-port oro-nasal thermistor, respiratory effort from continuous esophageal pressure (PES Sensor, Gaeltec CTO-1) and piezo-electric belts (Resp-EZ, EPM Systems, Midlothian, VA, USA), finger pulsoximetry (Embla A10 flex Sensor), electrocardiograms (V5), and a body position sensor.

Arterial samples for the analysis of blood gas parameters were taken in the supine position.

All the recordings were scored manually. An obstructive apnea was defined as the cessation of airflow for at least 10 seconds with continuing abdominal and thoracic movements, according to the American Academy of Sleep Medicine (18). An obstructive hypopnea was defined as a 50% reduction in airflow for at least 10 seconds, compared with baseline, accompanied by abdominal, thoracic, and esophageal pressure movements in combination with an arousal or an oxygen desaturation of 3% or more (18). Central apneas were defined as a cessation of airflow for 10 seconds without esophageal pressure fluctuations and respiratory movements. Sleep was scored manually in 30-second epochs according to Rechtschaffen and Kales (19). The obstructive apnea-hypopnea index (AHI) was defined as the mean number of obstructive apneas and hypopneas per hour of sleep, while the central apnea index was defined as the mean number of central apneas per hour of sleep. Obstructive sleep apnea was defined as obstructive apnea-hypopnea index of 5 or more. Central sleep apnea was defined as central and mixed apnea-hypopnea index of 5 or more.

The Epworth Sleepiness Scale (ESS) was used to assess the degree of daytime sleepiness. The ESS is a self-administered, validated questionnaire with eight questions relating to the risk of falling asleep in different situation, with answers scoring from 0 to 3. Possible scores ranged from 0 to 24, and excessive daytime sleepiness was defined as an ESS score of ≥11 (20).

### Statistical analysis

Continuous variables were given as the means ± standard deviation or medians and interquartile range (IQR). Categorical variables were presented as percentages. A paired *t* test was used for comparing normally distributed variables, and Wilcoxon’s signed rank test was used when comparing non-normally distributed variables. Proportions were compared using Fisher’s exact test or McNemar’s test for paired proportions. A *P* value of <0.05 was considered significant. Statistical calculations were performed with SPSS v 22 (SPSS Inc., Chicago, IL, USA).

## Results

Twenty-three patients, 14 men and 9 women, were investigated before and after the cardioversion of atrial fibrillation (Table 1). They were 62 ± 7 years old, with a mean BMI of 27 ± 4 kg/m^2^, and 19 patients (83%) had sleep apnea (AHI ≥5 events/h). Obstructive sleep apnea (obstructive AHI ≥5 events/h) occurred in 17/23 patients (74%) and central sleep apnea (central AHI ≥5 events/h) in 6/23 patients (26%). Five patients had both obstructive and central sleep apnea. Twenty-two patients had a normal or slightly impaired left ventricular function, with a left ventricular ejection fraction above 45%.

**Table 1.** Baseline characteristics.

	All (*n* = 23)	Sinus rhythm at follow-up (*n* = 16)	Atrial fibrillation at follow-up (*n* = 7)
Male, *n* (%)	14 (61)	10 (62)	4 (57)
Age (years)	62 ± 7	62 ± 8	61 ± 4
Body mass index (kg/m^2^)	27 ± 4	28 ± 4	30 ± 5
Hypertension, *n* (%)	12 (52)	8 (50)	4 (57)
Diabetes mellitus, *n* (%)	5 (21)	3 (19)	2 (29)
Ischemic heart disease, *n* (%)	2 (9)	1 (6)	1 (14)
Stroke, *n* (%)	1 (4)	0 (0)	1 (14)
Heart failure, *n* (%)	1 (4)	0 (0)	1 (14)
Echocardiography			
Normal or slightly impaired (LVEF >45%), *n* (%)	22 (96)	16 (100)	6 (86)
Moderately impaired (LVEF 30%–44%), *n* (%)	1 (4)	0 (0)	1 (14)
Medication (baseline)			
Beta-blockers, *n* (%)	17 (83)	13 (81)	14 (86)
Calcium antagonists, *n* (%)	6 (26)	4 (25)	2 (29)
Digoxin, *n* (%)	5 (18)	4 (25)	1 (14)
ACE inhibitors or ARB, *n* (%)	11 (48)	7 (44)	4 (57)
Diuretics, *n* (%)	7 (30)	4 (25)	3 (43)
Warfarin, *n* (%)	23 (100)	16 (100)	7 (100)
Statins, *n* (%)	7 (39)	3 (19)	4 (57)
Apnea-hypopnea index (AHI), events/h	24 ± 16	23 ± 16	28 ± 17
Obstructive AHI, events/h	18 ± 14	18 ± 15	18 ± 17
Central AHI, events/h	6.3 ± 14	4.8 ± 12	10 ± 17

Data are presented as means ± standard deviation for continuous variables, or numbers and percentages for dichotomous variables.

ACE: angiotensin-converting enzyme; AHI: apnea-hypopnea index; ARB: angiotensin II receptor blocker; LVEF: left ventricular ejection fraction.

**Figure 1.**
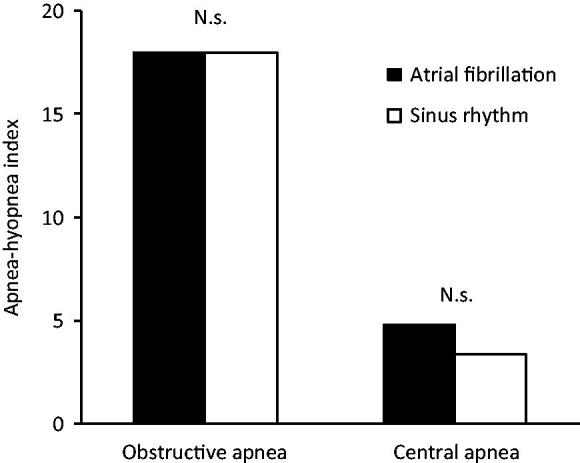
Mean obstructive and central apnea-hypopnea index among the 16 of 23 patients who were in atrial fibrillation at baseline and in sinus rhythm at follow-up.

Twenty-one patients were converted to sinus rhythm, and 16 of the 23 patients (70%) were in sinus rhythm at follow-up. Among the 16 patients who were in sinus rhythm at follow-up the first polysomnography was performed median 5.5 days (IQR 2–11.2) before cardioversion and the second polysomnography median 4 days (IQR 2–9.2) after cardioversion, and sleep apnea occurred in 13 (81%) before cardioversion and in 14 (88%) after cardioversion (*P* = 1.0) (Table 2). Obstructive sleep apnea occurred in 11/16 (69%) patients before cardioversion, and in 13/16 (81%) who were in sinus rhythm at follow-up (*P* = 0.5). Central sleep apnea occurred in 2/16 (12%) patients before cardioversion, and in 3/16 (19%) who were in sinus rhythm at follow-up (*P* = 1.0). Neither the obstructive apnea-hypopnea index (mean AHI 18 ± 15 versus 18 ± 14 events/h, *P* = 0.569) nor the central apnea-hypopnea index (mean AHI 4.8 ± 12 versus 3.4 ± 6.9 events/h, *P* = 0.593) changed when sinus rhythm was restored among patients who were in sinus rhythm at follow-up (Table 2, Figure 1). The proportion of patients with central sleep apnea at baseline was higher among those who had a recurrence of atrial fibrillation at follow-up (57% versus 12%, *P* = 0.045).

**Table 2.** Sleep apnea and sleep in subjects who had atrial fibrillation at baseline and sinus rhythm at follow-up.

	Baseline: atrial fibrillation (*n* = 16)	Follow-up: sinus rhythm (*n* = 16)	*P* value
Sleep apnea (AHI ≥5 events/h), *n* (%)	13 (81)	14 (88)	1.0
Obstructive sleep apnea, *n* (%)	11 (69)	13 (81)	0.5
Central sleep apnea, *n* (%)	2 (12)	3 (19)	1.0
Apnea-hypopnea index (AHI), events/h	23 ± 16	21 ± 14	0.918
Obstructive AHI, events/h	18 ± 15	18 ± 14	0.569
Central AHI, events/h	4.8 ± 12	3.4 ± 6.9	0.593
Total sleep time (TST) (min)	382 ± 67	378 ± 100	0.857
Sleep efficiency (%)	77 ± 15	79 ± 24	0.644
Stage 1 (% of TST)	14 ± 6.7	15 ± 8.7	0.339
Stage 2 (% of TST)	55 ± 12	55 ± 10	0.988
Stage 3 (% of TST)	11 ± 7.5	12 ± 7.7	0.640
REM (% of TST)	19 ± 7.9	18 ± 7.1	0.293
Supine position (% of TST)	27 ± 24	33 ± 19	0.397
Epworth Sleepiness Scale	7.7 ± 5.2	6.7 ± 5.0	0.216

Data are presented as means ± standard deviation for continuous variables, or numbers and percentages for dichotomous variables.

AHI: apnea-hypopnea index; central sleep apnea: central apnea-hypopnea index ≥5 events/h; obstructive sleep apnea: obstructive apnea-hypopnea index ≥5 events/h; TST: total sleep time.

Total sleep time, sleep in different sleep stages, sleep efficiency, sleep in a supine position, and daytime sleepiness according to the Epworth Sleepiness Scale did not change when sinus rhythm was restored (Table 2).

## Discussion

Sleep apnea was common in the present patients with atrial fibrillation, and 83% of the included patients had sleep apnea, although none of them had been diagnosed with sleep apnea previously. Obstructive sleep apnea occurred in 74% and central sleep apnea in 26% of our patients. At baseline, central sleep apnea was more common among patients with recurrence of atrial fibrillation at follow-up. The prevalence and the degree of sleep apnea were not affected by cardioversion. Sleep quality was normal at baseline and remained unaffected after the cardioversion.

The high prevalence of obstructive sleep apnea in the present patients with atrial fibrillation is close to the findings by Braga et al. and Albuquerque et al. (15,16). Obstructive sleep apnea is a known risk factor for cardiovascular disease, including stroke and hypertension (10,12,21–23). The suggested mechanisms of apnea-induced cardiovascular disease include hypoxia, increased sympathetic activity, and rapid changes in cerebral circulation when a subject struggles for air during obstructive apnea (24–26). Cadby et al. recently reported, in a prospective study, that obstructive sleep apnea is an independent risk factor for atrial fibrillation with an odds ratio of 1.55 (95% CI 1.21–2.00) (17). The high prevalences of obstructive apneas in patients with atrial fibrillation, and the fact that abolition of atrial fibrillation did not reduce obstructive apneas, further support the belief that obstructive sleep apnea is a risk factor for atrial fibrillation but not the opposite (5,15,16,27).

Central sleep apnea also occurs in patients with atrial fibrillation (16,28). In the absence of congestive heart failure, Leung et al. observed a high prevalence of atrial fibrillation also among patients with idiopathic central sleep apnea (28). The combination of congestive heart failure and atrial fibrillation has been suggested as a factor behind central sleep apnea (13,28). Reduced cardiac output and enhanced sensitivity to carbon dioxide induce respiratory system instability, which in turn trigger central sleep apnea (9,14,29,30).

Fox et al. recently reported that sleep apnea was reduced after restoration of sinus rhythm in 116 patients with atrial fibrillation (31). This reduction was due to a significant decrease in patients with central sleep apnea. We had expected that central apneas would be reduced when concomitant atrial fibrillation was converted into sinus rhythm. However, we had only few patients with central apnea, and we cannot conclude about any effect on central sleep apnea. We investigated fewer patients than Fox et al. Instead we used polysomnography including esophageal pressure monitoring, which is more specific than polygraphy, the method used by Fox et al. (31). The differences in methodology and sample size can explain the different results.

Other studies have addressed the question of whether restoring sinus rhythm reduces sleep apnea or not. Naruse et al. reported a decrease in the obstructive apnea-hypopnea index among 25 patients after radiofrequency catheter ablation of atrial fibrillation (32). They, however, only investigated patients with sleep apnea at baseline and not the whole cohort, which introduces a risk for regression towards the mean because of selection bias. Lissel et al. investigated only six patients with atrial fibrillation and observed no effect on the apnea-hypopnea index after cardioversion (33). Hoyer et al. reported that 74% of 23 patients with atrial fibrillation had sleep-disordered breathing before pulmonary vein isolation, with no change after the treatment of atrial fibrillation (27). They used simplified recordings and were unable to differentiate central from obstructive apneas. As opposed to the above studies, we investigated a whole cohort of patients before and after the cardioversion of atrial fibrillation.

A strength of the present study was the use of polysomnography including EEG, and esophageal pressure monitoring, the gold standard to distinguish central from obstructive apneas and hypopneas. A limitation was the low number of included patients. A major limitation was the even lower number of patients with central sleep apnea, and we can therefore not exclude an effect of cardioversion on central apneas.

In conclusion, both central and obstructive sleep apneas are common in patients with persistent atrial fibrillation. Obstructive sleep apneas are unaffected by the cardioversion of atrial fibrillation. The sleep pattern is normal and unaffected by cardioversion in patients with atrial fibrillation.
